# Scaled free-energy based reinforcement learning for robust and efficient learning in high-dimensional state spaces

**DOI:** 10.3389/fnbot.2013.00003

**Published:** 2013-02-28

**Authors:** Stefan Elfwing, Eiji Uchibe, Kenji Doya

**Affiliations:** Neural Computation Unit, Okinawa Institute of Science and Technology, Graduate UniversityOkinawa, Japan

**Keywords:** reinforcement learning, free-energy, restricted Boltzmann machine, robot navigation, function approximation

## Abstract

Free-energy based reinforcement learning (FERL) was proposed for learning in high-dimensional state- and action spaces, which cannot be handled by standard function approximation methods. In this study, we propose a scaled version of free-energy based reinforcement learning to achieve more robust and more efficient learning performance. The action-value function is approximated by the negative free-energy of a restricted Boltzmann machine, divided by a constant scaling factor that is related to the size of the Boltzmann machine (the square root of the number of state nodes in this study). Our first task is a digit floor gridworld task, where the states are represented by images of handwritten digits from the MNIST data set. The purpose of the task is to investigate the proposed method's ability, through the extraction of task-relevant features in the hidden layer, to cluster images of the same digit and to cluster images of different digits that corresponds to states with the same optimal action. We also test the method's robustness with respect to different exploration schedules, i.e., different settings of the initial temperature and the temperature discount rate in softmax action selection. Our second task is a robot visual navigation task, where the robot can learn its position by the different colors of the lower part of four landmarks and it can infer the correct corner goal area by the color of the upper part of the landmarks. The state space consists of binarized camera images with, at most, nine different colors, which is equal to 6642 binary states. For both tasks, the learning performance is compared with standard FERL and with function approximation where the action-value function is approximated by a two-layered feedforward neural network.

## 1. Introduction

Reinforcement learning (Sutton and Barto, 1998) has been proven to be effective for a wide variety of delayed reward problems. However, standard reinforcement learning algorithms cannot handle high-dimensional state spaces. For standard action-value function approximators, such as tile coding and radial basis function networks, the number of features of the function approximator grows exponentially with the dimension of the state- and action spaces.

Sallans and Hinton (2004) proposed free-energy based reinforcement learning (FERL) to handle high-dimensional state- and action spaces. In their method, the action-value function, *Q*, is approximated as the negative free-energy of a restricted Boltzmann machine (Smolensky, 1986; Freund and Haussler, 1992; Hinton, 2002). In this study, we propose a scaled version of FERL to achieve more robust and more efficient learning. The action-value function is approximated as the negative free-energy, divided with a constant scaling factor that is related to the size of the Boltzmann machine (the square root of the number of state nodes in this study). The initialization of the network weights and, thereby the initial *Q*-values, is a difficult problem in FERL. Even if the network weights are randomly initialized using a distribution with zero mean, the magnitude of the initial free-energy grows with the size of the network. The introduction of a scaling factor can, therefore, reduce this problem by initializing the *Q*-values to a more appropriate range. In addition, the scaling of the free-energy reduces the effect of a change in the weight values (i.e., a learning update) on the approximated *Q*-values. This makes it less likely that the learning diverges or get trapped in suboptimal solutions.

To validate the scaled version of FERL, we compare the learning performance with standard FERL and learning with a two-layered feedforward neural network. Our first experiment is a digit floor gridworld task, where the states are represented by images of handwritten digits from the MNIST data set. The purpose of the task is to investigate our proposed method's ability to extract task-relevant features in the hidden layer, i.e., to cluster images of the same digit and to cluster images of different digits that correspond to states with the same optimal action. We also test the method's robustness with respect to different exploration schedules, i.e., different settings of the initial temperature and the temperature discount rate in softmax action selection. Our second experiment is a robot visual navigation task, where the goal is to reach the correct goal area, which can be inferred by the color of the upper part of four landmarks. The color of the lower part of each landmark is unique and identifies the landmark's position, and can therefore be used for localization.

Apart from Sallans' and Hinton's (Sallans and Hinton, 2004) pioneering work, there have been few studies using a free-energy approach to function approximation in reinforcement learning. In our earlier study (Elfwing et al., 2010), we demonstrated the feasibility to use FERL for on-line control with high-dimensional state inputs in a visual navigation and battery capturing task with similar experimental setup as the visual navigation task in this study. We also demonstrated successful on-line learning in a real robot for a simpler battery capturing task. In this study, we compare the performance of scaled FERL with the standard FERL approach that was used in our earlier study. Otsuka et al. (2010) extended the FERL method to handle partially observable Markov decision processes (POMDPs), by incorporating a recurrent neural network that learns a memory representation that is sufficient for predicting future observations and rewards. The incorporation of memory capability does not improve the learning performance of standard FERL for the MDP tasks considered in this study.

## 2. Method

### 2.1. Gradient-descent Sarsa(λ)

The FERL method that we propose here is based on the on-policy reinforcement learning algorithm (Sutton and Barto, 1998) Sarsa(λ) (Rummery and Niranjan, 1994; Sutton, 1996), which learns an estimate of the action-value function, *Q*^π^, while the agent follows policy π. If the approximated action value function, *Q*_*t*_ ≈ *Q*^π^, is parameterized by the parameter vector θ_*t*_, then the gradient-descent update of the parameters is

(1) $$\theta_{t\;+\;1}=\theta_{t}+\alpha \delta_{t}e_{t},$$

where the TD-error, δ_*t*_ is

(2) $$\delta_{t}=r_{t}+\gamma Q_{t}(s_{t\;+\;1},a_{t\;+\;1})-Q_{t}(s_{t},a_{t}),$$

and the eligibility trace vector, *e*_*t*_, is

(3) $$e_{t}=\gamma \lambda e_{t\;-\;1}+\nabla_{\theta_{t}}Q_{t}(s_{t},a_{t}),\;e_{0}=0.$$

Here, *s*_*t*_ is the state at time *t, a*_*t*_ is the action selected at time *t, r*_*t*_ is the reward for taking action *a*_*t*_ in state *s*_*t*_, α is the learning rate, and γ is the discount factor of future rewards, λ is the trace-decay rate, and ∇_θ_*t*__*Q*_*t*_ is the vector of partial derivatives of the function approximator with respect to each component of θ_*t*_. In this study, the action-value function is approximated by the negative free-energy of a restricted Boltzmann machine.

### 2.2. Free-energy based function approximation

**Figure 1:**
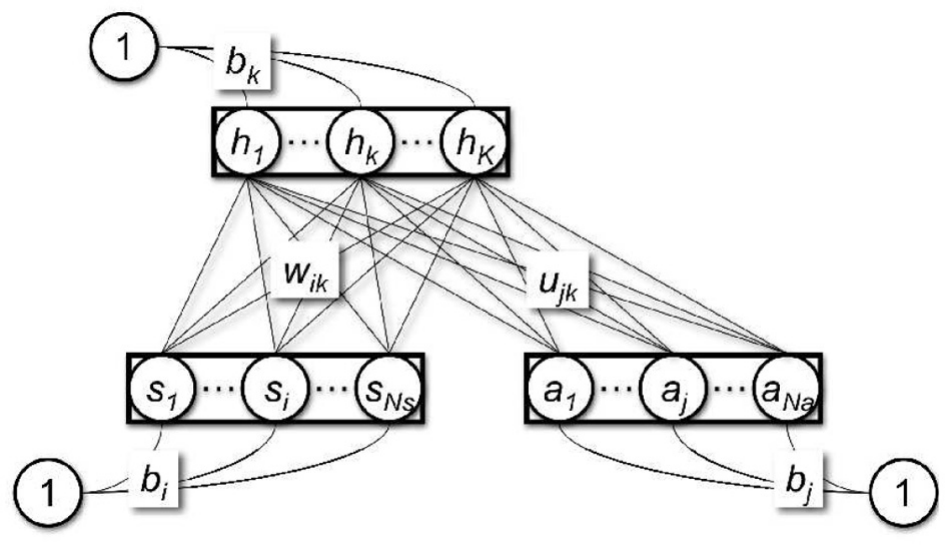
**Restricted Boltzmann machine**.

The use of a restricted Boltzmann machine (Smolensky, 1986; Freund and Haussler, 1992; Hinton, 2002) as a function approximator for reinforcement learning was proposed by Sallans and Hinton (2004). A restricted Boltzmann machine (Figure 1) is a bi-directional neural network which consists of binary state nodes, *s*, binary action nodes *a*, and hidden nodes, *h*. The *i*th state node, *s*_*i*_, is connected to hidden node *h*_*k*_ by the weight *w*_*ik*_, and the *j*th action node, *a*_*j*_, is connected to hidden node *h*_*k*_ by the weight *u*_*jk*_. In addition, the state nodes, the action nodes, and the hidden nodes are all connected to a constant bias input with a value of 1, with connection weights *b*_*i*_, *b*_*j*_, and *b*_*k*_, respectively. The free-energy, *F*, of the restricted Boltzmann machine is given as

(4) $$\begin{array}{l} F(s,a)=-\sum_{k=1}^{K}\left(\sum_{i=1}^{N_{s}}w_{ik}s_{i}h_{k}+\sum_{j=1}^{N_{a}}u_{jk}a_{j}h_{k}\right)-\sum_{i=1}^{N_{s}}b_{i}s_{i}\\ \;-\sum_{j=1}^{N_{a}}b_{j}a_{j}-\sum_{k=1}^{K}b_{k}h_{k}+\\ \;+\sum_{k=1}^{K}\left(h_{k}\log h_{k}+(1-h_{k})\log(1-h_{k})\right). \end{array}$$

Here, *K* is the number of hidden nodes, *N*_*s*_ is the number of state nodes, and *N*_*a*_ is the number of action nodes. The free-energy of each action *j* is computed by setting the corresponding action node, *a*_*j*_, to 1 and the rest of the action nodes to 0. *h*_*k*_ is the activation of the *k*th hidden node, given as

(5) $$h_{k}=\sigma \left(\sum_{i=1}^{N_{s}}w_{ik}s_{i}+\sum_{j=1}^{N_{a}}u_{jk}a_{j}+b_{k}\right),$$

where

(6) $$\sigma (x)=\frac{1}{1+e^{-x}}.$$

In Sallans' and Hinton's (Sallans and Hinton, 2004) original proposal, the action-value function was approximated by the negative free-energy, i.e., *Q*_*t*_ = −*F*_*t*_. In this study, we propose that the performance and the robustness of free-energy based function approximation can be improved by scaling the free-energy by a constant scaling factor, *Z*, that is related to the size of the Boltzmann machine, i.e., *Q*_*t*_ = −*F*_*t*_/*Z*. The update of the learning parameters (Equations 1–3) then becomes

(7) $$\theta_{t+1}=\theta_{t}+\alpha \delta_{t}e_{t},$$

(8) $$\delta_{t}=r_{t}-\gamma \frac{F_{t}(s_{t+1},a_{t+1})}{Z}+\frac{F_{t}(s_{t},a_{t})}{Z},$$

(9) $$e_{t}=\gamma \lambda e_{t-1}+\frac{1}{Z}\nabla_{\theta_{t}}(-F_{t}(s_{t},a_{t})).$$

The derivatives of the negative free-energy, with respect to the function approximator parameters (*w*_*ik*_, *u*_*jk*_, *b*_*i*_, *b*_*j*_, and *b*_*k*_), can be computed as

(10) $$\begin{array}{l} \nabla_{w_{ik}}\left(-F(s,a)\right)=s_{i}h_{k},\\ \nabla_{u_{jk}}\left(-F(s,a)\right)=a_{j}h_{k},\\ \;\nabla_{b_{i}}\left(-F(s,a)\right)=s_{i},\\ \;\nabla_{b_{j}}\left(-F(s,a)\right)=a_{j},\\ \;\nabla_{b_{k}}\left(-F(s,a)\right)=h_{k}. \end{array}$$

Since

(11) $$\begin{array}{l} \theta_{t+1}=\theta_{t}+\alpha \left(r_{t}-\gamma \frac{F_{t}(s_{t+1},a_{t+1})}{Z}+\frac{F_{t}(s_{t},a_{t})}{Z}\right)\\ \;\sum_{i=1}^{t}\frac{\gamma^{t-i}\lambda^{t-i}}{Z}\nabla_{\theta_{i}}(-F_{i}(s_{i},a_{i})), \end{array}$$

(12) $$\begin{array}{l} =\theta_{t}+\frac{\alpha }{Z^{2}}\left(Zr_{t}-\gamma F_{t}(s_{t+1},a_{t+1})+F_{t}(s_{t},a_{t})\right)\\ \;\sum_{i=1}^{t}\gamma^{t-i}\lambda^{t-i}\nabla_{\theta_{i}}(-F_{i}(s_{i},a_{i})), \end{array}$$

the scaled version of FERL can be transformed to the original formulation by re-scaling the learning rate (α′ = α/*Z*^2^) and the magnitude of the reward function (*r*′_*t*_ = *Zr*_*t*_).

### 2.3. Action selection

In this study, we use softmax action selection with a Boltzmann distribution, where the probability to select action *a* in state *s* is defined as

(13) $$P(a|s)=\frac{\exp(Q(s,a)/\tau )}{\sum_{b}\exp(Q(s,b)/\tau )}.$$

Here, τ is the temperature that controls the trade-off between exploration and exploitation. In this study, we used hyperbolic discounting of the temperature and the temperature was decreased every episode *i*:

(14) $$\tau (i)=\frac{\tau_{0}}{1+\tau_{k}i}.$$

Here, τ_0_ is the initial temperature and τ_*k*_ controls the rate of discounting.

To transform the scaled version to the original formulation when using softmax action selection, the temperature has also to be re-scaled (τ′ = *Z*τ).

### 2.4. Digit floor gridworld task

**Figure 2:**
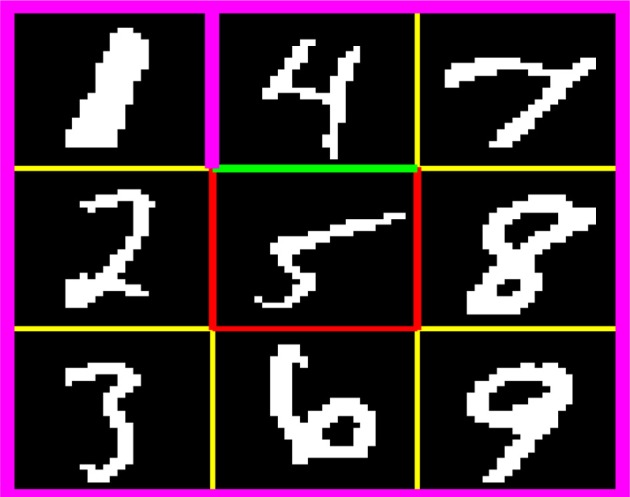
**Digit floor gridworld task.** The thick purple lines indicate the outer walls and the wall between states “1” and “4.” The yellow lines indicate zero reward state transitions. The red lines indicate negative reward (−0.01) for premature state transitions to the absorbing goal state (state “5”) from states “2,” “6,” and “8.” The green line indicates positive reward (+1) for successful completion of the task, i.e., state transition from state “4” to “5.”

Figure 2 shows the digit floor gridworld task. The thick purple lines indicate the outer walls and the wall between state “1” and state “4.” The yellow lines indicate zero reward state transitions. The red lines indicate negative reward (−0.01) for premature state transitions to the absorbing goal state (state “5”) from states “2,” “6,” and “8.” The green line indicates positive reward (+1) for successful completion of the task, i.e., state transition from state “4” to state “5.” There were four actions that moved the agent one step in the directions North, East, South, and West. If the agent moved into a wall, then the agent remained in the current state and received a zero reward. The agent started each episode at state “1” and the goal of the task was to reach state “5” by moving counterclockwise along a path through states “2,” “3,” “6,” “9,” “8,” “7,” and “4.” Each state consisted of an image of a handwritten digit from the MNIST data set (LeCun et al., 1998). The 28 × 28 pixels grayscale images were binarized by setting pixels with grayscale values larger than or equal to 128 to 1 and pixels with values smaller than or equal to 127 to 0. For each state, we used 20 different digit images that were randomly selected from the first 1000 images in the MNIST data set. At the start of each episode, the image for each state was randomly selected among the 20 possible images. An episode ended either when the agent moved to the absorbing state (state “5”) or after a maximum number of steps (set to 1000).

### 2.5. Robot visual navigation task

For the robot navigation task we used a simulation environment that was developed in MATLAB (2010) to mimic the properties of the Cyber Rodent robot (Doya and Uchibe, 2005). The Cyber Rodent is a small mobile robot, 22 cm in length and 1.75 kg in weight. The robot has a variety of sensors, including an omnidirectional C-MOS camera, an infrared range sensor, seven infrared proximity sensors, gyros, and accelerometers. It has two wheels and a maximum speed of 1.3 ms^−1^. In addition to an on-board CPU (SH-4), it has an FPGA for real-time color blob detection.

**Figure 3:**
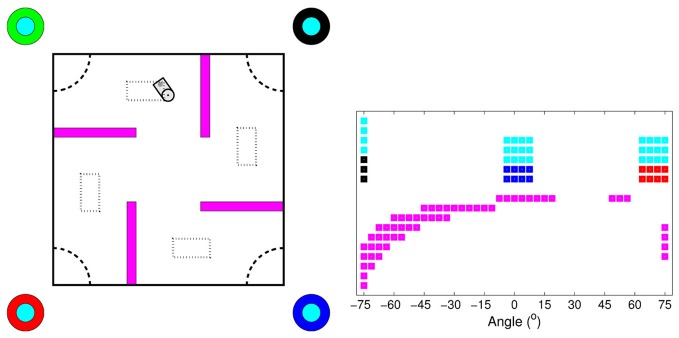
**Overview of the experimental area for the visual navigation tasks (left panel) and the camera image corresponding to the robot's position in the environment (right panel).** In the left panel, the dashed quarter circles at the corners indicate the four goal areas and the dotted rectangles indicate the starting areas. The circles outside the experimental area indicate the four landmarks. The color of the lower part of each landmark was unique and non-changing. The color of the upper part of all landmarks corresponded to the correct goal area and was randomly changed at the start of each episode. Note that the difference in radius between the lower and the upper part of the landmarks is only for illustrative purposes. In the experiments, both parts of the landmarks had the same radius.

The goal of the robot task (Figure 3) was to navigate to one of the four goal areas in the corners of the 2.5 × 2.5 m experimental area [dashed quarter circles in Figure 3 (left panel)], by learning to infer the correct goal area by the color of the upper part of four landmarks (cyan color in Figure 3). The landmarks were located outside the corners of the experimental area. The color of the lower part of each landmark was unique and non-changing (red, green, blue, and black colors in Figure 3), and could therefore be used for localization. At the start of each episode, the correct goal area was randomly changed, and, thereby, also the corresponding color of the upper part of the landmarks. The robot was randomly placed in one of the four starting areas [dotted rectangles in Figure 3 (left panel)]. The initial position within the starting area and the robot's initial heading angle were also randomly selected. We performed experiments with one goal area (southwest), two goal areas (southwest and northeast), three goal areas (southwest, southwest, and northeast), and all four goal areas.

The robot's simulated camera had a resolution of 738 (41 × 18) pixels covering a horizontal field of view of ±75°, with a 3.75° distance between the pixels. It could detect up to nine different colored objects: obstacles (purple in Figure 3), the lower part of the four landmarks (red, green, blue, and black in Figure 3), and one to four colors of the upper part of the landmarks (cyan in Figure 3), depending on the number of goals in the experiment. Within the field of view, the landmarks were visible from all distances and the obstacles were visible up to 2 m. The size of an object in the camera image increased with the inverse of the distance to the object. The state vector was constructed by creating a binary image of equal size to the original image for each color the robot could detect. The pixels that detected a colored object was extracted from the original image and the same pixels in the corresponding binary image was set to 1. All other pixels were set to 0. In addition, the state vector consisted of three normalized real-valued distance measures from the robot's front proximity sensors, located at −30°, 0°, and +30° in relation to the robot's heading direction. The distance information was normalized to the interval [0, 1] and higher values corresponded to shorter distances. The total length of the state vector in the experiment with four goals was 6645 (41 × 18 × 9 + 3). The robot could execute five actions, pairs of velocities (cm/s) of the left and the right wheels: rotate right (20, −20), curve right (40, 20), go straight (30, 30), curve left (20, 40), and rotate left (−20, 20). Gaussian noise was added to each wheel velocity, with zero mean and a standard deviation equal to 1% of the amplitude of the velocity. An episode ended either when the robot moved its head inside the correct goal area or when the length of the episode exceeded a fixed threshold of 2000 time steps. The robot received a +1 reward if it reached the correct goal area, otherwise the reward was set to 0.

## 3. Results

To evaluate the proposed scaled version of FERL, we compared the performance with standard FERL and with function approximation using a two-layered feedforward neural network (hereafter NNRL). The state nodes *s*_*i*_ of the neural network were connected to *K* hidden nodes by weights *w*_*ik*_. The hidden nodes had sigmoid activation functions (Equation 6), δ_*k*_ = σ(∑_*i*_*w*_*ik*_*s*_*i*_). The hidden nodes were connected to *Q*-value output nodes with linear activation by weights *w*_*ka*_. The approximated *Q*-values were computed as the linear combination of the output weights and the hidden activation [*Q*(*s, a*) = ∑_*k*_*w*_*ka*_δ_*k*_], with derivatives with respect to the weight parameters computed as

(15) $$\begin{array}{l} \nabla_{w_{ik}}\left(Q(s,a)\right)=\delta_{k}(1-\delta_{k})w_{ka}s_{i},\\ \nabla_{w_{ka}}\left(Q(s,a)\right)=\delta_{k}. \end{array}$$

For the scaled FERL, we concluded after a trial and error process that a scaling factor equal to the square root of the number of state nodes ($Z=\sqrt{N_{s}}$) was an appropriate value for the experiments conducted in this study. For both tasks, the number of hidden nodes (*K*) was set to 20 for all three methods. In the gridworld task, we tested the robustness of the methods with the respect to different exploration schedules by comparing the learning performance for action selection with τ_0_ set to 0.5, 1, and 2 and τ_*k*_ set to 0.01, 0.001, and 0.0005 (Equation 14). In the robot navigation task, τ_0_ and τ_*k*_ were determined by searching for appropriate values in the experiment setting with two goal targets. τ_0_ was set to 0.5 for all three methods. τ_*k*_ was set to 0.01 for scaled FERL and 0.002 for FERL and NNRL. Table 1 shows the settings of α, γ, and λ in the experiments. For all three methods, the weights were randomly initialized using a Gaussian distribution with zero mean. For the weights connecting the state nodes and the hidden nodes the variance was equal to 0.001 and for weights connecting the hidden nodes and the action nodes the variance was equal to 1.

**Table 1:** **Meta-parameter settings for the experiments**.

	**Gridworld task**			**Robot task**		
	**Scaled FERL**	**FERL**	**NNRL**	**Scaled FERL**	**FERL**	**NNRL**
α	0.01 × Z	0.001	0.001	0.01 × Z	0.001	0.001
γ	0.96	0.96	0.96	0.98	0.98	0.98
λ	0.8	0.8	0.8	0.8	0.8	0.8

### 3.1. Digit floor gridworld task

**Figure 4:**
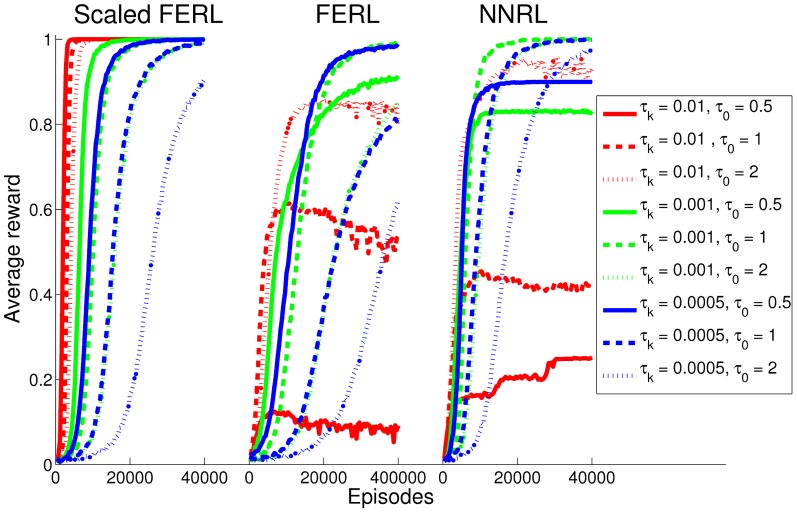
**The average reward computed over every 100 episodes and 20 simulation runs, for scaled FERL (left panel), FERL (middle panel), and NNRL (right panel).** The line colors correspond to the settings of τ_*k*_ (red: 0.01, green: 0.001, and blue: 0.0005) and the line types correspond to the setting of τ_0_ (solid: 0.5, dashed: 1, and dotted: 2).

For the gridworld task, we performed 20 simulations runs for each method and each setting of τ_0_ and τ_*k*_. Figure 4 shows the average rewards computed over every 100 episodes. The result clearly shows better and more robust learning performance for scaled FERL (left panel in Figure 4). The learning converged to average reward values exactly equal to, or close to equal to, the maximum reward of 1 for 8 out of the 9 different settings of τ_0_ and τ_*k*_. The only exception was the experiment with the largest initial temperature (τ_0_ = 2) and lowest discount rate (τ_*k*_ = 0.0005) where the average reward was still increasing at the end of learning (dotted blue line in the left panel in Figure 4). The learning speed was, not surprisingly, determined by the exploration schedule. Experiments with smaller initial temperatures and higher discount rates converged faster. In the experiment with the smallest initial temperature (τ_0_ = 0.5) and highest discount rate (τ_*k*_ = 0.01), the average learning performance reached close to 1 after about 2500 episodes (solid red line in the left panel in Figure 4). The learning then converged after about 5250 episodes with the average reward exactly equal to 1 with 0 variance. If we define successful learning as a simulation run where, at the end of learning, the greedy action [argmax_*a*_ *Q*(*s, a*)] was equal to the optimal action for all 20 digit images for all states, then scaled FERL was successful in 100% (20) of the simulation runs for eight settings of τ_0_ and τ_*k*_. The only exception was, again, the experiment with τ_0_ = 2 and τ_*k*_ = 0.0005, where 90% (18) of the simulation runs were successful.

**Figure 5:**
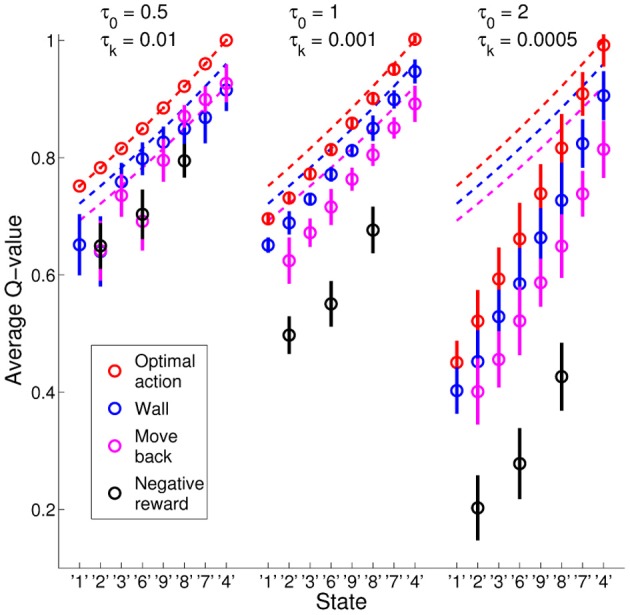
**Average *Q*-values (circles) with standard deviation (bars) computed over all 20 simulation runs using scaled FERL in the experiments with τ_0_ = 0.01 and τ_*k*_ = 0.01 (left panel), τ_0_ = 1 and τ_*k*_ = 0.001 (middle panel), and τ_0_ = 2 and τ_*k*_ = 0.0005 (right panel).** The figure shows the average learned *Q*-values, along the optimal path from the initial state “1” to the goal state “5,” of the four types of actions: (1) optimal actions (red); (2) actions that moved the agent into a wall (blue); (3) actions that moved the agent away from the goal (purple); and (4) negative rewarded actions (black).

The exploration schedule did also effect the learning of the *Q*-values for optimal and non-optimal actions. Figure 5 shows the average learned *Q*-values (circles) with standard deviations (bars) in the experiments with τ_0_ = 0.01 and τ_*k*_ = 0.01 (left panel), τ_0_ = 1 and τ_*k*_ = 0.001 (middle panel), and τ_0_ = 2 and τ_*k*_ = 0.0005 (right panel), computed over all state images for all states in all 20 simulation runs for scaled FERL. The different colors show the values of the four different types of actions for the states along path from the initial state “1” to the goal state “5”: (1) red for optimal actions; (2) blue for actions that moved the agent into a wall; (3) purple for actions that moved the agent away from the goal; and (4) black for negative rewarded actions. Since the goal reward was set to +1, the optimal *Q*-values (dashed red lines) were equal to γ^*t* − 1^, where *t* is the number of steps to the goal. A move into a wall increased the steps to the goal by one (optimal *Q*-values equal to γ^*t*^, see dashed blue lines) and actions that moved agent away from the goal increased the steps to the goal by 2 (optimal *Q*-values equal to γ^*t* + 1^, see dashed purple lines). In the experiment with the smallest initial temperature and highest discount rate (left panel in Figure 5), scaled FERL learned almost perfect *Q*-values for the optimal actions. For the non-optimal actions, the average *Q*-values differed significantly from the optimal *Q*-values and for several states the learned values were in the wrong order. This is explained by the fast convergence of the learning. The average number of steps to goal converged close to the optimal number of steps of 8 after about 5000 episodes and to exactly 8 steps after about 25,000 episodes. After the initial learning phase, there was almost no exploration to improve the estimates of the *Q*-values of the non-optimal actions, only exploitation of the already learned optimal actions. In the experiments with larger initial temperatures and lower discount rates (middle and right panels in Figure 5), scaled FERL not only learned estimates of the *Q*-values for the optimal actions, but of the full action-value function. In both experiments, there were clear separations between the average *Q*-values for all actions in all states. At the end of learning, there was still considerable exploration of the environment, even if the greedy actions were equal to the optimal actions for all, or almost all, state images. The average number of steps to the goal were 10.2 steps (τ_0_ = 1 and τ_*k*_ = 0.001) and 19.8 steps (τ_0_ = 2 and τ_*k*_ = 0.0005). The results showed a trade-off between fast learning convergence, which required fast decay of the temperature, and learning of the full action-value function, which required slower decay of the temperature and much longer learning time.

FERL and NNRL (middle and right panels in Figure 4) required careful tuning of both τ_0_ and τ_*k*_ to converge to average reward values close to the maximum reward of 1 within the learning time. FERL achieved this for only two settings of τ_0_ and τ_*k*_ (dashed green and solid blue lines in Figure 4) and NNRL achieved this for three settings (dashed green, dotted green, and dashed blue lines in Figure 4). The low average learning performance for many settings of τ_0_ and τ_*k*_ was caused by that the learning completely failed in some simulation runs. The agent either moved prematurely to the goal state (−0.01 reward), or the agent remained in the gridworld until the maximum number of steps (1000) had passed. In general, NNRL learned faster and had a higher rate of successful learning, compared with FERL. For NNRL, the highest rate of successful learning was 100% of the simulations runs (τ_0_ = 1 and τ_*k*_ = 0.001) and the average success rate, computed over all nine settings of τ_0_ and τ_*k*_, was 76%. For FERL, the highest success rate was 70% (τ_0_ = 1 and τ_*k*_ = 0.001) and the average success rate was only 30%.

**Figure 6:**
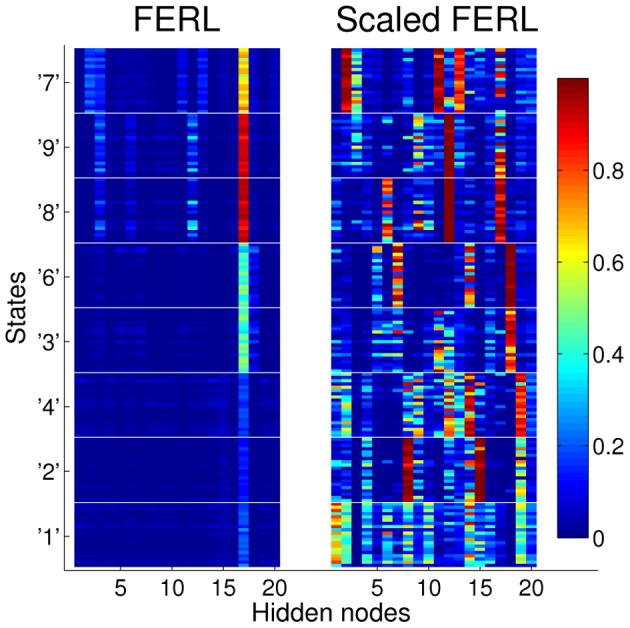
**Typical hidden activation patterns after successful learning for FERL (left panel) and scaled FERL (right panel).** The figures show the activation of all 20 hidden nodes for all 20 digit images for all states.

To try to explain the difference in performance between the scaled version of FERL and standard FERL, we looked at the patterns of activation in the hidden nodes. Figure 6 shows typical hidden activation patterns after successful learning, for all 20 digit images for all states. The displayed activation patterns are grouped according to state and optimal action, i.e., South for states “1,” “2,” and “4,” East for states “3” and “6,” North for states “8” and “9,” and West for state “7.” The difference in hidden activation patterns between the two methods is quite remarkable. FERL learned a very sparse and strong action-coding with minimal separation between images of the same digit and between states with the same optimal action. The action-coding was achieved with a few active hidden nodes and the majority of the nodes were silent for all state inputs. In the hidden activation pattern shown in Figure 6, the action-coding was achieved using almost only hidden node 17. The actions were separated by differences in the node's activation level: 0.16 ± 0.04 for South, 0.44 ± 0.06 for East, 0.92 ± 0.03 for North, and 0.65 ± 0.06 for West. In contrast, the coding learned by scaled FERL was much more complex with no silent hidden nodes. The pattern of hidden activation did not only separate states according to optimal action, there was also clear differentiation between states and even individual state images.

### 3.2. Robot visual navigation task

**Figure 7:**
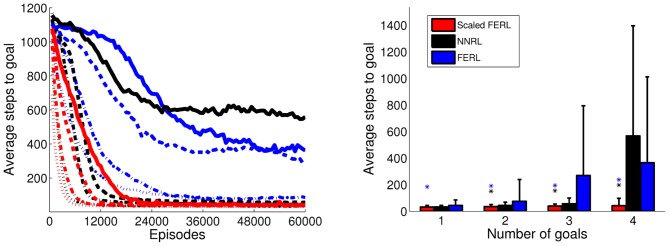
**The average number of time steps to goal for the whole learning process (left panel) and in the final 100 episodes (right panel), for the four experiments with 1, 2, 3, and 4 goal areas.** The average values were computed over every 100 episodes and 10 simulation runs in each experiment. In the left panel, the line type indicates the number of goals: dotted lines for 1 goal, dash-dotted lines for 2 goals, dashed lines for 3 goals, and solid lines for 4 goals. The colored asterisks in the right panel indicate experiments in which the final average performance of scaled FERL was significantly better (*p* < 0.001) than NNRL (black) or FERL (blue).

The result of robot navigation task is summarized in Figure 7. The left panel shows the average number of steps to goal, computed over every 100 episodes and 10 simulation runs for each experiment. The right panel shows the average number of steps to goal with standard deviation in the final 100 episodes. Scaled FERL converged to similar average number of time steps to goal, with low variance, in all simulation runs in each of the four experiments. The learning converged faster and the final learning performance was significantly better (*p* < 0.001) in all four experiments. The only exception was NNRL in the one goal experiment, which performed very similar to scaled FERL, both with respect to convergence speed and final learning performance. For experiments with 2 and 3 goals, NNRL performed almost as well as scaled FERL. The learning performance decreased significantly in the experiment with four goals. NNRL failed to learn to navigate to the goal for at least one starting area and one goal area in 7 (out of 10) simulation runs. The final learning performance of FERL was reasonably good in the experiments with one and two goals. The learning only failed in one simulation run, in the experiment with two goals. However, the convergence speed was slow compared to the other two methods. In the experiments with 3 and 4 goals, the learning performance decreased significantly and the learning failed in 4 and 5 simulation runs, respectively.

**Figure 8:**
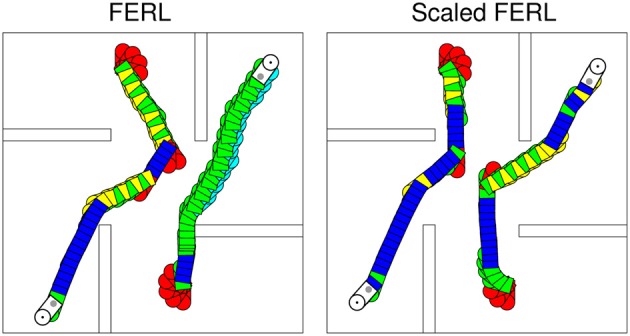
**Typical trajectories learned by FERL (left panel) and scaled FERL (right panel) for navigating to the northeast goal and the southwest goal in the experiment with two goals.** The color coding indicates the selected actions: red for rotate right (action 1), green for curve right (action 2), blue for go straight (action 3), yellow for curve left (action 4), and cyan for rotate left (action 5).

**Figure 9:**
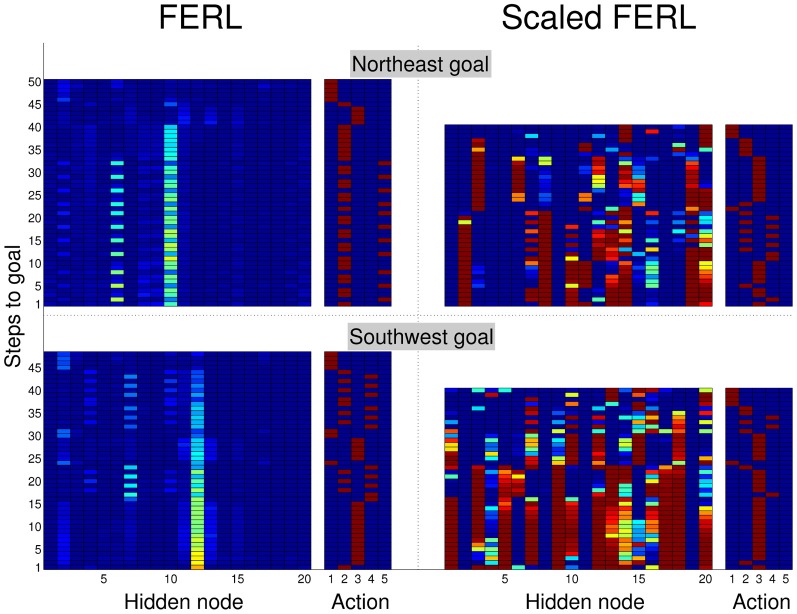
**Hidden activation patterns and selected actions for the states along the trajectories to the northeast goal (top panels) and southwest goal (bottom panels) shown in Figure 8, for FERL (left panels) and scaled FERL (right panels)**.

To try to explain the difference in learning performance between standard FERL and scaled FERL, we looked at learned trajectories and the corresponding hidden activation patterns. Figure 8 shows typical trajectories learned by FERL (left panel) and scaled FERL (right panel) for navigating to the northeast (NE) goal and the southwest (SW) goal in the experiment with two goals, starting from the center of the south starting area and the north starting area, respectively, and facing the outer wall. The color coding indicates the selected actions: red for rotate right (action 1), green for curve right (action 2), blue for go straight (action 3), yellow for curve left (action 4), and cyan for rotate left (action 5). Figure 9 shows the activation of the hidden nodes and the selected actions along the learned trajectories.

The learned policies and the hidden activation patterns were very different between the two methods. FERL learned a policy which selected separate combinations of actions for navigation to different goal areas. In the example shown in the left panel in Figure 8, the robot only executed the curve right and the rotate left actions to reach the NE goal, after the initial part of the trajectory. To reach the SW goal, the robot executed either the curve right and the curve left actions to pass obstacles, or the go straight action to move toward the goal and the rotate right action for course corrections. FERL learned, as in the gridworld task, a very sparse and strong action-coding with little separation between states corresponding to the same action (left panels in Figure 9). Each action corresponded to the activation of one or few hidden nodes, e.g., hidden node 6 coded action 5 (rotate left) and hidden node 7 coded action 4 (curve left). Scaled FERL learned a policy which selected similar actions in corresponding positions along the trajectories to different goals, as shown in the right panel in Figure 8. In contrast to FERL, there was clear differentiation in the hidden activation patterns for different states (right panels in Figure 9).

## 4. Discussion

In this study, we proposed a scaled version of FERL, where the action-value function is approximated as the negative free-energy of a restricted Boltzmann machine, divided by a constant scaling factor. The scaling factor was set to the square root of the number of state nodes. To validate our proposed method, we compared the learning performance with standard FERL and with NNRL (function approximation using a two-layered feedforward neural network), for a digit floor gridworld task and a robot visual navigation task. The learning with scaled FERL performed significantly better than the other two methods for both tasks. In the gridworld task, we also compared the robustness with respect to different exploration schedules (i.e., settings of initial temperature and temperature discount rate in softmax action selection). The learning with scaled FERL was very robust and the results showed a trade-off between fast learning convergence, which required fast decay of the temperature, and learning of the full action-value function, which required slower decay of the temperature and much longer learning time. In contrast, the learning with FERL and NNRL could only converge to average reward values close to the maximum reward for a narrow range of initial temperatures and discount rates. Analysis of activation patterns in the hidden nodes showed big differences between FERL and scaled FERL. FERL learned a very sparse action-coding with little separation between different states corresponding to the same action. In contrast, scaled FERL learned a much richer neural encoding with no silent hidden nodes and clear separation between different states corresponding to the same action.

Although quite arbitrary, the setting of the scaling factor to the square root of the number of state nodes worked very well for the tasks considered in this study. One reason was probably that we used the same number of hidden nodes (20) in all experiments. A more general setting of the scaling factor should probably also include the number of hidden nodes, because the magnitude of the initial negative free-energy increases with the number of hidden nodes of the Boltzmann machine. For example, in the gridworld task, the magnitude of the initial negative free-energy is about 16 with 20 hidden nodes, about 80 with 100 hidden nodes, and about 160 with 200 hidden nodes. An alternative approach would be to include the scaling factor as a parameter of the function approximator. The scaling factor, *Z*, would then be updated according to ∇_*Z*_*Q*_*t*_ = *F*_*t*_/*Z*^2^. We plan to investigate the setting of the scaling factor more thoroughly in future work.

The introduction of the scaling factor can ensure that the *Q*-values are initialized within a more appropriate range, e.g., between zero and one in the episodic delayed reward tasks with a goal reward of +1 considered in this study. This could partly explain why the learning with scaled FERL was more stable than learning with FERL. However, it does not explain the much faster convergence speed of scaled FERL and the remarkable difference in activation patterns of the hidden nodes. These issues will also be explored in future work.

In our earlier research, we have developed methods such as multiple model-based reinforcement learning (MMRL) (Doya et al., 2002) and competitive-cooperative-concurrent reinforcement learning with importance sampling (CLIS) (Uchibe and Doya, 2004) to improve the learning performance and the learning speed of reinforcement learning. FERL and such methods are complementary and suitable for different types of learning tasks. Restricted Boltzmann machines are global function approximators. They grow linearly with number of nodes and they are, therefore, well suited for tasks with very high-dimensional binary state inputs, such as binarized images. FERL offers few, if any, benefits in tasks with low-dimensional state spaces and real-valued state input. MMRL has proven to work well for low-dimensional non-linear control problems, but would, in our opinion, not scale well to tasks with very high-dimensional state input. In addition, MMRL requires a continuous reward function, because each module learns its policy in separate parts of the state space and there is no sharing of values between modules. In the two task in this study, it would therefore be impossible for a module to learn a policy for a part of the trajectory to the goal, since the reward is zero for all state transitions except transitions to the absorbing goal state. CLIS was developed for tasks with real-valued state input. CLIS selects an appropriate policy out of a set of heterogeneous modules with different levels of resolution in the state representation (i.e., simpler modules with coarse discretization of the state input and more complex modules with fine discretization of the state input). The CLIS framework, therefore, offers no benefit for tasks with binary state inputs. A common alternative approach to use an advanced function approximator, such as FERL, is to use a hybrid approach with a separate state abstraction module combined with a simple reinforcement learning algorithm. In our experience, a hybrid approach makes concurrent learning difficult, because it in most cases requires pre-training of the state abstraction module to achieve efficient learning. The experimental results in this study show that scaled FERL can achieve both fast learning convergence (with appropriate settings of τ_0_ and τ_*k*_) and generalization of the state space in the neural encoding in the hidden layer.

In this study, we used a machine learning approach to visual navigation in neurorobotics, where the neural encoding is an emergent property of the function approximation used in the learning algorithm. An alternative approach is to use biologically-inspired computational modeling of the brain circuits involved in navigation in real animals (Arleo and Gerstner, 2000; Krichmar et al., 2005; Fleischer et al., 2007; Barrera and Weitzenfeld, 2008; Giovannangeli and Gaussier, 2008; Milford and Wyeth, 2010; Caluwaerts et al., 2012). Currently, the two approaches are mostly complementary. In the former approach, the main focus is to develop efficient and robust learning algorithms that works well for a wide variety of learning tasks. In the latter approach, the main focus is to increase our understanding of the underlying brain mechanisms of animal behavior. The most important test is whether the robot's behavior and the activity of the simulated nervous system match empirical data from experiments with real animals. A natural long-term goal of neurorobotics would be to merge the two approaches to achieve both efficient learning and biologically plausible neural encoding.

### Conflict of interest statement

The authors declare that the research was conducted in the absence of any commercial or financial relationships that could be construed as a potential conflict of interest.
